# Electroconvulsive therapy-induced volumetric brain changes converge on a common causal circuit in depression

**DOI:** 10.21203/rs.3.rs-2925196/v1

**Published:** 2023-06-01

**Authors:** Zhi-De Deng, Olga Theresa Ousdal, Leif Oltedal, Brian Angulo, Mate Baradits, Andrew Spitzberg, Ute Kessler, Alexander Sartorius, Annemiek Dols, Katherine Narr, Randall Espinoza, Jeroen Van Waarde, Indira Tendolkar, Philip van Eijndhoven, Guido van Wingen, Akihiro Takamiya, Taishiro Kishimoto, Martin Jorgensen, Anders Jorgensen, Olaf Paulson, Antoine Yrondi, Patrice Peran, Carles Soriano-Mas, Narcís Cardoner, Marta Cano, Linda van Diermen, Didier Schrijvers, Jean-Baptiste Belge, Louise Emsell, Filip Bouckaert, Mathieu Vandenbulcke, Maximilian Kiebs, Rene Hurlemann, Peter Mulders, Ronny Redlich, Udo Dannlowski, Erhan Kavakbasi, Michael Kritzer, Kristen Ellard, Joan Camprodon, Georgios Petrides, Anil Maholtra, Christopher Abbott, Miklos Argyelan

**Affiliations:** NIMH-NIH; Department of Clinical Medicine, University of Bergen; Department of Clinical Medicine, University of Bergen; Department of Clinical Medicine, University of Bergen; Department of Clinical Medicine, University of Bergen; Department of Clinical Medicine, University of Bergen; Department of Clinical Medicine, University of Bergen; Central Institute of Mental Health; Geffen School of Medicine at the University of California, Los Angeles; Geffen School of Medicine at the University of California, Los Angeles; Departments of Neurology, Psychiatry and Biobehavioral Sciences, University of California; Rijnstate; Donders Institute for Brain, Cognition and Behavior, Department of Psychiatry; Donders Centre for Cognitive Neuroimaging; Amsterdam UMC, University of Amsterdam; Keio University School of Medicine; Keio University School of Medicine; Psychiatric Center Copenhagen; Psychiatric Center Copenhagen; Rigshospitaler & University of Copenhagen; Unité ToNIC, UMR 1214 CHU PURPAN -; Unité ToNIC, UMR 1214 CHU PURPAN -; IDIBELL; Institut d’Investigació Biomèdica Sant Pau (IIB-Sant Pau), Hospital de la Santa Creu i Sant Pau. Centro de Investigación Biomédica en Red de Salud Mental, Instituto de Sa; UPC KU Leuven; UPC KU Leuven; UPC KU Leuven; UPC KU Leuven; UPC KU Leuven; UPC KU Leuven; KU Leuven, University Psychiatric Center KU Leuven; Institute for Translational Psychiatry, University of Münster; Institute for Translational Psychiatry, University of Münster; Institute for Translational Psychiatry, University of Münster; Institute for Translational Psychiatry, University of Münster; Institute for Translational Psychiatry, University of Münster; Massachusetts General Hospital, Harvard Medical School; Massachusetts General Hospital, Harvard Medical School; Massachusetts General Hospital, Harvard Medical School; Massachusetts General Hospital, Harvard Medical School; Zucker Hillside Hospital; Zucker Hillside Hospital; University of New Mexico School of Medicine; Feinstein Institutes for Medical Research

## Abstract

Neurostimulation is a mainstream treatment option for major depression. Neuromodulation techniques apply repetitive magnetic or electrical stimulation to some neural target but significantly differ in their invasiveness, spatial selectivity, mechanism of action, and efficacy. Despite these differences, recent analyses of transcranial magnetic stimulation (TMS) and deep brain stimulation (DBS)-treated individuals converged on a common neural network that might have a causal role in treatment response. We set out to investigate if the neuronal underpinnings of electroconvulsive therapy (ECT) are similarly associated with this common causal network (CCN). Our aim here is to provide a comprehensive analysis in three cohorts of patients segregated by electrode placement (N = 246 with right unilateral, 79 with bitemporal, and 61 with mixed) who underwent ECT. We conducted a data-driven, unsupervised multivariate neuroimaging analysis (Principal Component Analysis, PCA) of the cortical and subcortical volume changes and electric field (EF) distribution to explore changes within the CCN associated with antidepressant outcomes. Despite the different treatment modalities (ECT vs TMS and DBS) and methodological approaches (structural vs functional networks), we found a highly similar pattern of change within the CCN in the three cohorts of patients (spatial similarity across 85 regions: r = 0.65, 0.58, 0.40, df = 83). Most importantly, the expression of this pattern correlated with clinical outcomes. This evidence further supports that treatment interventions converge on a CCN in depression. Optimizing modulation of this network could serve to improve the outcome of neurostimulation in depression.

## Introduction

One of the oldest and most effective forms of neurostimulation is electroconvulsive therapy (ECT)^1,2^ However, despite the last decades of ECT-neuroimaging research, its mechanism of action is not known. In a recently published article, Siddiqi et al. ^3^ showed that a common underlying neural network (Supplementary Fig. 1A) is associated with the clinical response of treatment resistant depression in transcranial magnetic stimulation (TMS) and deep brain stimulation (DBS). Additionally, dysfunctions within this network explain clinical symptoms in patients with stroke, multiple sclerosis and other forms of brain lesions ^3,4^. These results are not mere associations, but instead indicate that interference within this network could explain individual differences in treatment response. The main cortical areas associated with the common causal network included regions previously implicated in depression and emotion regulation, such as the subgenual cingulate cortex, dorsolateral prefrontal cortex, ventromedial prefrontal cortex, inferior frontal gyrus, frontal eye field, and intraparietal sulcus (Supplementary Fig. 1A, ^3,5^).

While ECT is not a localized form of treatment, the applied ECT-induced electric field (EF) has unique spatial distribution specific to an individual and the electrode placement ^6–9^. High frequency electric field (EF) stimulation has a direct neuroplastic effect on the brain ^10–12^ and is also associated with downstream biological effects through the induced seizure activity ^13,14^. In agreement with these preclinical findings, recent large-scale studies in the Global ECT-MRI Research Collaboration (GEMRIC) dataset ^15^ found robust volume increases ^16,17^ in a wide range of cortical and subcortical regions, which correlated with the number of ECT sessions. Subsequent EF modeling based on the individual head MRI consistently demonstrated that the ECT-induced EF strongly correlated with volume increase ^18–20^. These results verified that despite the widespread activation of the brain through seizure, the direct electrical stimulation effect of ECT is much more spatially selective and individually diverse than first assumed.

Despite these replicable and robust structural findings driven by the spatial distribution of the EF, their direct or indirect effect on clinical outcome remain unclear. Univariate analysis of the EF amplitude on clinical response was the subject of several previous investigations. However, the results were somewhat contradictory ^18,21,22^. Similarly, the robust changes in volume did not translate into correlations with clinical response in most of the studies ^16,17^ or indicated a relationship where volume increase in the dentate gyrus was associated with worse clinical outcome ^23^.

One caveat was the primarily univariate nature of these analyses. The brain regions are not independent of each other, and multivariate analysis could be more sensitive to detect network-wide changes ^3^. Indeed, one follow-up analysis of the GEMRIC dataset in 192 individuals with supervised multivariate models could detect networks of regions where the weighted average of the changes correlated with clinical outcomes ^24^. The results of this analysis showed that the linear combination of volume changes across 18 regions correlated with clinical outcomes. The loadings of the 18 regions showed some similarities with the spatial distribution of the causal map published by Siddiqi et al. (Supplementary Fig. 1B, r = 0.33, df = 16). Although these 18 regions comprise a limited coverage of the causal map, it raises the intriguing possibility that the ECT-induced volume changes might follow a similar spatial pattern already described with functional connectivity analysis in other treatment modalities such as TMS and DBS.

To address this question, we revisit and improve the analysis of EF-structure to clinical outcomes by doubling the sample size across three independent cohorts (total N = 386). We implement an unsupervised learning algorithm (Principal Component Analysis, PCA) running separately on EF and structural data, and separately on different electrode placements (6 parallel PCAs). The non-supervised learning methods reduce the risk of overfitting. Any convergence in these independent but parallel multivariate analyses would strongly support the validity of our findings and indicate a common pathway in the mechanism of action of ECT. Finally, we will demonstrate not only that a common circuit emerges, but that it is remarkably similar to the causal circuit previously published in the context of TMS and DBS efficacy ^3^.

## Methods

### Participants

386 ECT-treated subjects were analyzed from the GEMRIC consortium ^15^. This multi-site consortium collects data in a centralized server from ECT-treated patients who underwent longitudinal neuroimaging and clinical assessment. The 386 subjects were recruited at 19 sites and their respective demographics and clinical data are in Supplementary Table 1. All contributing sites received ethics approval from their local ethics committee or institutional review board. In addition, the centralized mega-analysis was approved by the Regional Ethics Committee South-East in Norway (No. 2013/1032).

### Volume changes

The image processing methods have been detailed previously ^16–18^. In brief, the sites provided longitudinal 3T T1-weighted MRI images (at baseline and after the end of the course of ECT) with a minimal resolution of 1.3 mm in any direction. The raw DICOM images were uploaded and analyzed on a common server at the University of Bergen, Norway. To guarantee reproducibility, in addition to the common platform, the processing pipelines were implemented in a docker environment ^25^. First, images were corrected for scanner-specific gradient-nonlinearity ^26^. Further processing was performed with FreeSurfer version 7.1, which includes segmentation of subcortical structures ^27^ and automated parcellation of the cortex ^28^. In addition to brainstem and bilateral cerebellum, this automated process identified 33 cortical and eight subcortical regions in each hemisphere. Altogether this resulted in 85 regions of interest (ROIs) (Supplementary Tables). Next longitudinal FreeSurfer analysis was used for unbiased, within-subject assessment of estimation of longitudinal volume change (ΔVol - %) (Supplementary Fig. 3). In more detail, we cross-sectionally processed both time points separately with the default FreeSurfer workflow and created an unbiased template from both time points for each subject. Once this template is created, parcellations and segmentation are carried out at each time point initialized with common information from the within-subject template ^29^. In summary, we calculated bias-free estimation of volumetric change from 85 brain regions across the timespan of an ECT course in 386 individuals who received on average of 12.5 ± 5.4 ECT sessions.

### EF modeling

Our approach was detailed in one of our previous manuscripts ^18^, with the upgraded software of Roast 3.0 (Realistic Volumetric-Approach to Stimulate Transcranial Electric Stimulation v3.0) ^6^. In short, ROAST builds a three-dimensional tetrahedral mesh model of the head based on the T1 MRI images of the brain. Then, segmentation identifies five tissue types: white and gray matter of the brain, cerebrospinal fluid, skull, and scalp, and assigns them different conductivity values: 0.126 S/m, 0.276 S/m, 1.65 S/m, 0.01 S/m, and 0.465 S/m respectively. ECT electrodes of 5 cm diameter were placed over the C2 and FT8 EEG (10–20 system) sites to model RUL, and over to FT8 and FT9 sited to model BT electrode placements. Study sites from the GEMRIC database used either the Thymatron (Somatics, Venice, Florida) or spECTrum (MECTA Corp., Tualatin, Oregon) devices. EF was solved using the finite-element method with unit current on the electrodes and, subsequently, it was scaled to the current amplitude of the specific devices (Thymatron 900 mA, spECTrum 800 mA). We had 61 individuals who had to switch from RUL to BT electrode placement during the ECT course. This is a standard clinical practice in patients with inadequate clinical response with RUL stimulation. In these cases, we calculated the EF with the weighted mean according to the number of ECT sessions the individual had in each form of placements. For example, if a patient had 6 ECTs with RUL and then had 18 ECTs with BT then we calculated 0.25 × EF_RUL_ + 0.75 × EF_BT_ in each region. These procedures resulted in a voxel-wise EF distribution map in each individual. We calculated the average EF across the 85 three-dimensional ROIs at baseline in every individual based on the Freesurfer parcellations and segmentations. The voxel values with the top and lowest one percentile in each ROI were omitted during calculations to reduce boundary effects.

### Multivariate analysis

To investigate the regional volume changes and EF amplitudes in a multivariate way, we applied principal component analysis (PCA). We conducted six consecutive PCA analyses on RUL, BT, and MIX separately for EF and structural data, respectively (variables were normalized across individuals before PCA). We separated the groups as we wanted to avoid capturing differences that were only electrode placement specific. We used Cattell’s scree test to determine the number of PCs to analyze. We found that the first 2 PCs captured most of the variance, and the subsequent PCs captured a diminishing portion of the variance (elbow criteria, Supplementary Fig. 2). We conducted posthoc analyses to evaluate 1) the correlation between PCs and clinical outcomes: ΔMADRS ~ PC1 + PC2 + age + nECT (nECT: number of ECT sessions, ΔMADRS: percent change compared to baseline (T2-T1)/T1, negative values indicate better response), and 2) the spatial similarity between loadings and the CCN ^3^. To investigate if one hemisphere was driving the results, we conducted the PCA separately on the right and left hemisphere (Supplementary Material).

### Covariates

We conducted multivariable regression analysis to estimate the effect of the calculated principal components on clinical response. This analysis included the principal components of the volume change, EF, age and number of ECT sessions as independent variables. These last two variables were included as confounders. As it is explained below, age correlated with EF and clinical response, and number of ECT sessions were also correlating with clinical response and volume change. Therefore, these variables had to be added to correct for spurious correlations.

### Justification of the confounding variables

We corrected for two variables consistently across our analyses. We would like to provide a brief justification for including these. We also provide a causal model with a corresponding directed acyclic causal graph to illustrate the reasoning (Supplementary Fig. 7).

### 1. Number of ECT sessions

It was already noted in the first large scale publication of the GEMRIC consortium that the number of ECT sessions and clinical response correlated in a counterintuitive way: the larger the number of ECT sessions registered between MRI assessments, the lower the clinical response was. The explanation of this observation is that most of the participating sites in the GEMRIC consortium acquired the follow-up (post-ECT) MRI after completing the (un-) successful ECT course, in contrast to predetermined length of treatment period with a fixed number of ECT-sessions. This resulted in an earlier timepoint of post-ECT MRI assessment if there was a quick clinical response, but later when clinical improvement was delayed or absent. This is problematic because the number of ECT sessions positively correlates with the volume change during ECT (dose–response effect). Therefore, not controlling for the number of ECT sessions can easily lead to spurious correlations indicating that volume increase was associated with worse outcome, or just simply mask the otherwise real effect when volume change is beneficial. Indeed, in recent cohorts where the length of ECT course between the neuroimaging sessions were predetermined, authors found positive relationships between hippocampus volume increase and clinical response ^19^ (Table 1).

### 2. Age

Our sample had a tight correlation between age and clinical response as well. This correlation is typical in ECT datasets ^30–32^, as the elderly patients respond to ECT significantly better. This introduces, however, another confound to every EF modeling as age negatively correlates with EF magnitude in the human brain due to structural changes such as atrophy ^9^. This age and EF relationship was particularly strong in RUL (right unilateral) placement (R Hippocampus; RUL: r=−0.31, p < 0.001, df = 244, BT: r=−0.17, p = 0.13, df = 77, MIX: r=−0.28, p = 0.03, df = 59), therefore it could mask the effect of EF on clinical response in our previous analysis ^18^.

The code relevant to this manuscript is available at https://github.com/argyelan/Publications/tree/master/VOLUME-CHANGE-PCA.

## Results

### EF correlates with volume changes

**Figure 1:**
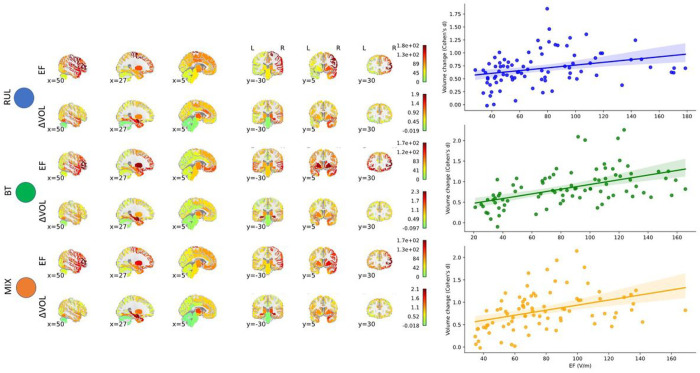
Electric field (EF) and volume change (ΔVOL) correlate across 85 regions in RUL (blue), BT (green) and MIX (orange) electrode placements. The upper line shows the average EF (V/m), the lower line shows the effect size of the volume change (Cohen’s d) color coded. The right panel shows the values in the corresponding regions in a scatterplot.

The dataset is detailed in Supplementary Table 1A and B. Like our previous studies, we found volume increases in almost every region across the brain with effect sizes (Cohen’s d) ranging from − 0.02 to 1.93, corresponding 0% (Left Cerebellum) to 6.7% (Right Amygdala) volume increases. 75% of the 85 regions had a volume increase of at least 0.5 effect size or higher (t > 9.8, p < 10^− 12^, df = 385, Supplementary Table 2A). 246 patients received right unilateral (RUL) ECT placement only, 79 bitemporal (BT) only, and 61 individuals first started with RUL and were later switched to BT (MIX) treatment. The overall volume changes were higher in the BT and MIX groups than in the RUL group (mean volume increase: 3.5% ± 1.7%, 3.4% ± 2.1% vs 1.7% ± 1.9%). The BT and MIX had larger number of ECT sessions on average (RUL: 10.9 ± 4.4; BT: 14.3 ± 6.2; MIX: 16.4 ± 5.3) ^17^. In addition, independent of the number of ECT sessions, BT and MIX also had higher average EF amplitude in the brain (RUL: 49.0 ± 8.7 V/m; BT: 91.8 ± 15.1 V/m; MIX: 68.7 ± 13.4 V/m). Our results replicated our previous findings in patients with RUL placement ^18^, extended to other types of electrode placements, and demonstrated a strong correlation across the regions between average EF and volume change in all three groups separately and combined (Fig. 1, RUL: r = 0.30, p = 0.005; BT: r = 0.50, p = 8.5 × 10^− 7^, MIX: r = 0.39, p = 0.0002, df = 83). Several regions showed strong correlations between EF and volume change across individuals even if corrected for age and number of ECT sessions. In agreement with our previous study ^18^ left hippocampus and amygdala showed the strongest relationship (false discovery rate (FDR) corrected: L Hippocampus: t_EF_ = 7.03, p_FDR_ = 4 × 10^− 10^, Left Amygdala: t_EF_ = 9.18, p_FDR_ = 2×10^− 16^, Supplementary Table 3A). This relationship remained the same if we removed the 151 individuals with RUL who were the participants of the previous publication ^18^, (Supplementary Table 3B).

### Unsupervised multivariate analysis

**Figure 2:**
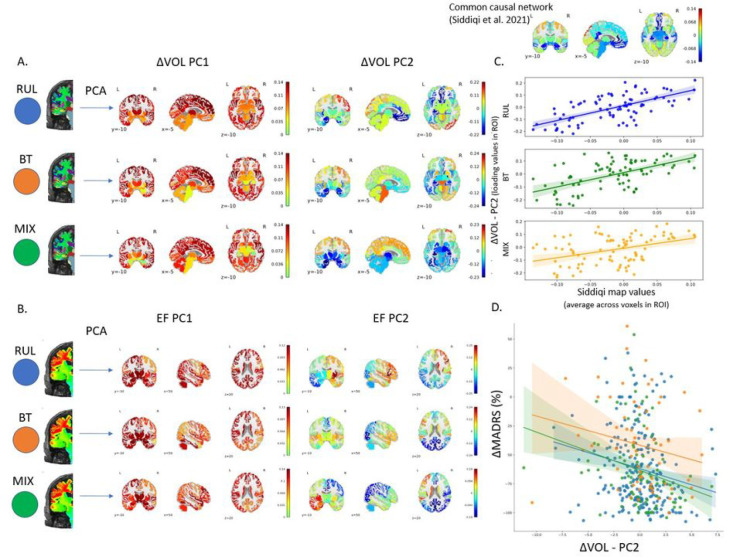
Unsupervised multivariate analysis (principal component analysis - PCA) of the regional volume changes (A) and the EF values (B). The first component (left side) reflected the main effect. The second PC of the volume change was very similar to the Common Causal Network recently reported by Siddiqi et al. (2021) regardless of the electrode placement (C). The second PC of the EF reflected the spatial distribution stemming from the different electrode placements. The second PC of the volume changes not only correlated with the map reported by Siddiqi et al, but also correlated with the clinical outcome (D), the more similar the pattern was to the Common Causal Network the better the clinical outcome was.

Volume changes: In agreement with previous findings ^17^ the first PC (PC1) (Fig. 2A left) was responsible for 42%, 42%, and 41% of the variance in the volume changes in the RUL, BT, and MIX groups, respectively. This 42% variance indicated a strong intra-individual cross-correlation in regional volume increase. The loadings of this main effect showed spatial similarity with the common causal network (RUL: r = 0.44, p = 2×10^− 5^; BT: r = 0.50, p = 1×10^− 6^, MIX: r = 0.46, p = 8×10^− 6^; df = 83) even though it was an unsupervised finding. The second PC (PC2) (Fig. 2A right) was responsible for 6%, 8%, and 11% of the variance and the loading was spatially very similar to the common causal network (RUL: r = 0.65, p = 2×10^− 11^; BT: r = 0.58, p = 6×10^− 9^, MIX: r = 0.40, p = 0.0002; df = 83, Fig. 2C). Moreover, the effect sizes of similarity of loadings of the second component were higher in all groups than in the first component.

### Spatial coordinates as covariates

We conducted a multiple regression of the “CCN values” ~ abs(X) + Y + Z, where X, Y, and Z were the coordinates across the LR (left-right), PA (posterior-anterior) and the IS (inferior-superior) axes, respectively. The Siddiqi map values showed strong correlations across the spatial dimensions of the regions, especially across the posterior-anterior and inferior-superior axis (F_3,81_ = 19.6, p = 1×10^− 9^; t_Xabs_ = 5.1, p < 0.0001, t_Y_ = −2.2, p = 0.03, t_Z_ = 5.2, p < 0.0001), indicating higher values on the lateral and on the superior areas. The solid spatial similarities between the CCN and the main (loadings of PC1) and the secondary effect (loadings of PC2) raised the question if these maps were only reflecting gross similarities across the posterior-anterior or inferior-superior direction. One could argue that both RUL and BT had a higher impact on the superior and lateral areas, resulting in a more reliable volume change in these regions leading to a PC that had grossly matching spatial distribution with the CCN. We tested this hypothesis by conducting a multiple regression with spatial coordinates of the regions as confounders: “CCN values” ~ PC1 + PC2 + X + Y + Z in all three groups (*RUL*: F_5,79_ = 19.07, p = 2×10^− 12^; t_PC1_ = 2.13, p = 0.04; **t**_**PC2**_**= 4.66, p = 1×10**^**− 5**^; t_Xabs_ = 2.25, p = 0.03; t_Y_ = 1.10, p = 0.27; t_Z_ = −0.38, p = 0.70, *BT*: F_5,79_ = 19.18, p = 2×10^− 12^; t_PC1_ = 2.07, p = 0.04; **t**_**PC2**_**= 4.37, p = 4×10**^**− 5**^; t_Xabs_ = 3.44, p = 0.0009; t_Y_ = −1.78, p = 0.08; t_Z_ = 0.84, p = 0.40, and *MIX*: F_5,79_ = 12.8, p = 4×10^− 9^; t_PC1_ = 1.15, p = 0.26; t_PC2_ = 0.69, p = 0.49; t_Xabs_ = 4.25, p = 6×10^− 5^; t_Y_ = −2.43, p = 0.02; t_Z_ = 2.32, p = 0.023). In both RUL and BT groups, the results equivocally identified that PC2 showed highly significant similarities that could not be explained by gross anatomical similarities (Supplementary Table 4A, B, and C).

Volume change PC2 and not PC1 correlates with clinical response: Critical to our investigation, our multiple regression analysis ΔMADRS ~ PC1_ΔVOL_ + PC2_ΔVOL_ + age + nECT indicated that PC2, with its remarkable similarity to the CCN, had a significant correlation with clinical response (F_4,381_ = 15.95, p = 5×10^− 12^; t_PC1_ = −0.51, p = 0.61; **t**_**PC2**_**= −2.35, p = 0.019**; t_age_ = −5.83, p < 0.0001; t_nECT_ = 2.96, p = 0.003, Fig. 2D). The more similar the volume change was with the PC2 the better the clinical outcome.

EF amplitude: The first PC (Fig. 2B left) was responsible for 70%, 65%, and 57% of the variance in the EF amplitude in the RUL, BT and MIX groups, respectively. This high variance in the first PC indicated a strong intra-individual correlation across the brain regions, meaning that individuals with high EF had higher EFs across different regions. Therefore, this first PC represented an overall EF magnitude across subjects, which was due to individual differences in brain and head anatomy, including the amount of cerebrospinal fluid and fat tissue. The second PC (Fig. 2B right) was responsible for 7%, 10%, and 24% of the variance, respectively. The spatial distribution of the second PC reflected the electrode placement, showing higher loading near the electrode locations. The loadings of PC2 did not show any significant correlation with the CCN in RUL and BT. In the MIX group, the PCA analysis indicated that the main (PC1) and electrode effect (PC2) was more interleaved, reflecting in the lower and higher variances in the first and second PC. This was also reflected in its loading structure. Overall, none of the PCs from the EF amplitudes showed any correlation with the CCN once it was corrected for the spatial coordinates (Supplementary Tables 4A, B and C).

EF amplitude PC1 and not PC2 correlates with clinical response: A multiple regression analysis ΔMADRS ~ PC1_EF_ + PC2_EF_ + age + nECT indicated that PC1, representing overall EF strength, had a significant correlation with clinical response (F_4,381_ = 15.55, p = 9×10^− 12^;**t**_**PC1**_**= 2.11, p = 0.036**; t_PC2_ = −0.19, p = 0.85; t_age_ = −4.97, p < 0.0001; t_nECT_ = 3.21, p = 0.001). The higher the EF amplitude in general was associated with inferior clinical response. PC1_EF_ and PC2_ΔVOL_ negatively correlates (r = −0.29, p = 6×10^− 9^, df = 384) across the individuals, implying that higher overall EF amplitude in the human brain was associated with lower expression of the PC2_ΔVOL_, which we established to be associated with good clinical effect. Multivariate analysis of ΔMADRS ~ PC1_EF_ + PC2_ΔVOL_ + age + nECT showed that while the effect of PC1_EF_ was not significant, the PC2_ΔVOL_ remained significant (F_4,381_ = 16.66, p = 1×10^− 12^; t_PC1EF_ = 1.64, p = 0.1; **t**_**PC2ΔVOL**_**= −1.96**, p = **0.05**; t_age_ = −4.97, p < 0.0001; t_nECT_ = 2.76, p = 0.006), indicating tighter association of the clinical outcome with ΔVOL. We complemented our analyses above by conducting linear mixed models by adding sex and electrode placement as fixed effects and site as a random effect, but it did not change the results (Supplementary Material).

### Laterality

Finally, we repeated the same multivariate analyses on the right and the left side of the brain independently (42 ROIs in each hemisphere). We found this necessary for two reasons. First, there is a large literature indicating differences among hemispheres in mood regulation ^33^. Second, both the map reported by Siddiqi et al. and the bilateral placement maps are highly symmetrical which could lead to a spurious overestimation of the results.

The loadings of the PCs acquired on the entire brain appeared highly symmetrical (Supplementary Fig. 4). As expected, the PCs of the EF in the RUL setting were the least symmetrical, but the volume changes were more symmetrical across different electrode placements. When we ran the PCAs separately for the right and left hemispheres (PCA_right_, PCA_left_), we found that the results were very similar for the PCA_right_ (Supplementary Figs. 5 and 6, r: 0.72–0.99). We found that the second PC of volume change highly correlated with the CCN and with the clinical response (Supplementary Material). The second PC of volume change also correlated highly with the loadings of the original PCA (Supplementary Fig. 6). The PCA_left_ led to a similar loading structure in their first PCs of the EF and volume changes (main effect, r = 0.61–0.95), but the second PCs were different in RUL and BT (r = 0.01, 0.47, respectively) and volume change did not correlate with clinical outcome (Supplementary material).

## Discussion

Our study is a comprehensive multivariate analysis of 386 patients with depression who underwent ECT and longitudinal neuroimaging. Our multivariate non-supervised analysis (PCA) revealed a hidden pattern in volume change that was correlated with clinical outcome. The same pattern was found independently in the three separate groups, RUL, BT and MIX electrode placements, and this pattern showed striking similarities to the common causal circuit recently published in a study of large cohort of independent samples of depressed patients ^3,4^. This network consists of cortical and sub-cortical areas previously implicated in depression or emotion regulation, such as the subgenual cingulate cortex, dorsolateral prefrontal cortex, ventromedial prefrontal cortex and hippocampus.

Initial studies of ECT effect on structural neuroimaging on limited sample sizes (N ~ 20) often focused on hippocampus increase. As it became clear later, more widespread volume changes with moderate effect sizes were present in these samples, but due to the limited sample size, it did not reach statistical significance ^15^. After establishing the GEMRIC consortium ^15^ and collecting hundreds of individuals, ECT studies repeatedly and consistently showed increased volume in both cortical and subcortical regions ^16–18^. The GEMRIC data also demonstrated that the volume increase correlated with the EF amplitude in RUL electrode placement ^18^. This relationship between EF and volume change was recently replicated in an independent cohort ^19^. The current findings further confirm this relationship in an array of groups with different and often mixed electrode placements (RUL, BT, and MIX). The previous findings were replicated, and the weighted mixing of the EF values according to the number of ECT sessions on different electrode placements proved to be a useful way to calculate the effect of EF on volume change (MIX electrode placement). Our current study further confirms that EF modeling, despite its limitation ^34^, is a useful technique to estimate EF, and the growing body of evidence of the correlation between EF and volume changes serves as biological validation of the technique.

### Multivariate analysis and clinical effect

We found a spatial pattern in the volume changes on top of the main effect which showed distinct similarities to the CCN map reported by Siddiqi et al and was responsible for approximately 6–11% of the total variance. Most importantly, the more this pattern was expressed, the better the clinical outcome was. Our approach had two vital aspects that could further boost confidence about the validity of these findings. First, it was unsupervised and data-driven, to avoid overfit and no information about the CCN was used to conduct our analysis. Second, we analyzed the three electrode placement groups separately as independent samples. We not only received equivalent results across distinct groups, but these results were highly similar to the common causal circuit reported by Siddiqi et al. As reported in patients treated with DBS and TMS, individual similarity to this map correlated with antidepressant outcome in ECT.

A set of interconnected areas, with sometime opposite signs in their relationship, is reliably implicated in association with depression. In addition to the original discovery ^3^, a very recent lesion mapping study in patients with multiple sclerosis resulted in a very similar map, showing close correlation with depression rates ^4^. While our methodology deviates from traditional lesion mapping, as it is measuring structural volume change patterns, the second principal component of the variance shows a distinct similarity with the networks reported previously. The brain wide volume changes which failed to associate with clinical response likely includes both epiphenomena and antidepressant volumetric changes ^17^. Our results suggest that the antidepressant volumetric changes are relatively hidden (PC2) behind the main volumetric effect of ECT (PC1). This explains why previous univariate approaches failed to detect correlations between volume change and clinical response. Without decomposing the volume changes to a main effect (PC1), which is responsible for most of the variance, and to an orthogonal pattern (PC2), this second pattern would remain hidden. Our results indicate that only this second pattern of change, which is similar to the pattern obtained by Siddiqi et al, has clinical relevance.

Furthermore, multivariate analysis of the electric field revealed two components: the first represented the main effect, the overall strength of the EF across the brain, and the second was specific to the spatial particularities of the electrode placement. The first component that reflected the overall EF strength correlated with clinical response, indicating that higher EF was associated with worse outcome. We also found that the higher the first component of EF was expressed, the lower the CCN expression in the patient (the second component of the volume change). In a classical sense ^35^ our multiple regression analysis between clinical effect and the principal components of the EF and ΔVOL might imply that the high EF was mediated through a lower expression of the beneficial pattern, leading to a less than optimal outcome. As there are several limitations to deduct causal inference from classical mediation analysis, we point out here the need of prospective studies with preselected parameters to determine true cause and effect relationships in the future. The observation that high general EF was associated with worse clinical outcome is counterintuitive first, but supported by one previous study in a smaller, and only in BT treated, cohort (Fridgeirsson et al. 2021), and might indicate that more focal or low amplitude treatments would be more beneficial. This seemingly contradicts some of the clinical observations in recent studies of RUL patients where the amplitude of the current was modified and was found that below certain range the clinical effect was insufficient ^22^. However, these tested values (600, 700 and 800 mA) were below the range we use in current clinical practice and constitute the database analyzed in this article (800 and 900 mA). These results suggest that too low or too high EF might be equally suboptimal (inverted U hypothesis of strength of EF and antidepressant outcomes) ^36^. The optimal strength of EF and its proper spatial distribution is an intriguing new direction that must be systematically investigated in the future.

Finally, PCA on each hemisphere independently showed that the first PC, representing the main effect, was similar regardless of the analytical approach (whole brain, right or left side, Supplementary Fig. 6). There were, however, hemispheric differences in the second PCs: whole-brain PCA results were only replicated on the right side. This implies that neuroplastic changes associated with clinical outcomes were more robust on the right side ^37,38^.

In summary, this current report is a significant step forward in understanding the direct electrical stimulation aspect of ECT and its effect on brain volume changes and clinical outcomes. Our deepening understanding in this domain could lead to informed decisions in designing novel studies and methods to optimize treatments. In addition, this study revealed that the same neural network associated with clinical benefits in TMS and DBS is implicated in ECT as well.
